# Primary undifferentiated sarcoma in the thorax: a rare diagnosis in young patients

**DOI:** 10.1590/0100-3984.2015.0165

**Published:** 2016

**Authors:** Carlos Henrique Simões de Oliveira Waszczynskyi, Marcos Duarte Guimarães, Luiz Felipe Sias Franco, Bruno Hochhegger, Edson Marchiori

**Affiliations:** 1 Hospital Heliópolis, São Paulo, SP, Brazil.; 2 A.C.Camargo Cancer Center e Hospital Heliópolis, São Paulo, SP, Brazil.; 3 Universidade Federal de Ciências da Saúde de Porto Alegre (UFCSPA), Porto Alegre RS, Brazil.; 4 Universidade Federal do Rio de Janeiro (UFRJ), Rio de Janeiro, RJ, Brazil.

Dear Editor,

A 30-year-old man was admitted to the thoracic surgery department of a tertiary hospital for investigation of a thoracic mass. Having previously received treatment for pneumonia, he presented with a two-week history of progressively increasing pain in the right hemithorax and right flank, between the anterior axillary line and midaxillary line. On clinical examination, there was an absence of breath sounds in the right hemithorax.

**Figure 1:**
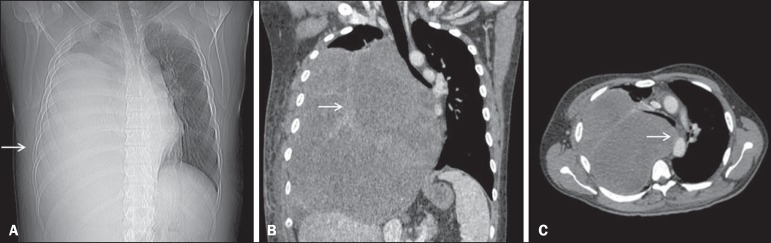
CT scan showing a primary sarcoma in the right hemithorax. **A:** CT scout image showing opacification of the right hemithorax. **B:** Coronal CT reconstruction with heterogeneous enhancement (arrow). **C:** Axial CT slice showing contralateral mediastinal deviation.

**Figure 2:**
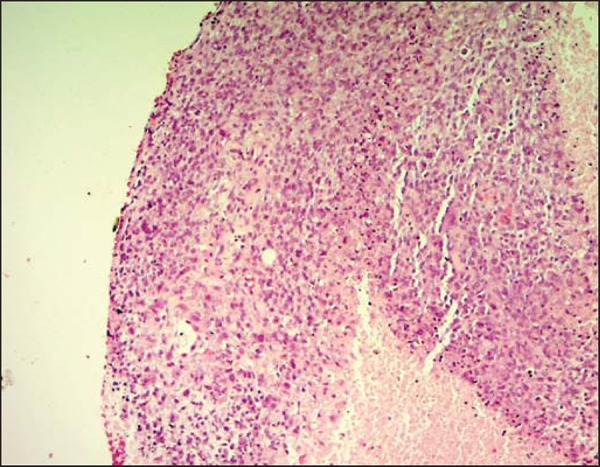
Undifferentiated sarcoma. Hematoxylin-eosin staining (×100).

Computed tomography (CT) of the chest showed an extensive, heterogeneous, mostly solid mass in right thoracic region (Figure 1), with areas of inner content of low attenuation (21-26 Hounsfield units) and foci of bleeding, without intervening calcifications and without osteolysis of the rib. Laboratory tests produced results within the limits of normality. The patient underwent percutaneous biopsy, and the pathology examination revealed undifferentiated sarcoma (Figure 2).

Sarcomas represent a heterogeneous group of tumors derived from mesenchymal cells^(1-3)^. They account for 1% of all neoplasms and occur mainly in the extremities (in 60% of cases), gastrointestinal tract (in 25%), retroperitoneal space (in 20%), and the head and neck region (in 4.1%). Primary sarcomas of the thorax are exceptionally rare, accounting for only 0.2% of lung cancers and only 5% of all the thoracic neoplasms. Such sarcomas can involve the lungs, mediastinum, pleura, and, mainly, the chest wall. The presence of sarcoma in any other part of the body must be ruled out, because metastasis to the chest is much more common than is primary sarcoma of the thorax^(4-7)^.

The most common histological types of primary sarcomas are angiomyosarcoma, leiomyosarcoma, rhabdomyosarcoma, and sarcomatoid mesothelioma^(8)^. In the chest wall, the most common primary sarcomas are Ewing's sarcoma, primitive neuroectodermal tumor, malignant fibrous histiocytoma, chondrosarcoma, osteosarcoma, synovial sarcoma, and fibrosarcoma^(8)^. Radiologically, these tumors typically present as large, heterogeneous masses. However, their appearance can vary from an intrabronchial mass to an intravascular mass or even a solitary pulmonary nodule^(8)^.

In the case reported here, the patient was young, had no comorbidities, and presented with a voluminous mass in the right intrathoracic right region, the initial diagnostic suspicion pointing to sarcoma.

In accordance with the literature, the analysis of clinical data and CT images obtained can only suggest primary sarcoma of the thorax as one of the differential diagnoses; the differentiation between sarcoma subtypes is only possible through pathological examination of the biopsy sample^(8)^.

Therefore, although it is a rare neoplasm, primary sarcoma must be considered among the diagnoses of thoracic tumors, especially when a large heterogeneous mass is identified in a young patient without evidence of malignancy in another part of the body.
